# Scleroglucan-Based Foam Incorporating Recycled Rigid Polyurethane Waste for Novel Insulation Material Production

**DOI:** 10.3390/polym16101360

**Published:** 2024-05-10

**Authors:** Luca Cozzarini, Lucia Marsich, Alessio Ferluga

**Affiliations:** 1 Department of Engineering and Architecture, University of Trieste, Via Valerio 10, I-34127 Trieste, Italy; 2 MaterialScan S.r.l., Via Capodistria 28, I-34145 Trieste, Italy; lmarsich@materialscan.it (L.M.); aferluga@materialscan.it (A.F.)

**Keywords:** thermal insulating material, soundproofing, rigid polyurethane recycling, biopolymer, circular economy

## Abstract

This study details the synthesis and performance evaluation of a novel lightweight thermal and acoustic insulation material, resulting from the combination of a scleroglucan-based hydrogel and recycled rigid polyurethane waste powder. Through a sublimation-driven water-removal process, a porous three-dimensional network structure is formed, showcasing notable thermal and acoustic insulation properties. Experimental data are presented to highlight the material’s performance, including comparisons with commercially available mineral wool and polymeric foams. This material versatility is demonstrated through tunable mechanical, thermal and acoustic characteristics, achieved by strategically adjusting the concentration of the biopolymer and additives. This adaptability positions the material as a promising candidate for different insulation applications. Addressing environmental concerns related to rigid polyurethane waste disposal, the study contributes to the circular economy.

## 1. Introduction

The control of temperature in buildings and enclosed spaces currently accounts for approximately 40% of global energy consumption [1,2,3]. Achieving proper thermal insulation and improving energy efficiency are critical steps in reducing heat flow and energy consumption, which in turn can help mitigate related emissions [4,5]. The insulating materials market is currently dominated by inorganic fibrous materials (glass and mineral wool) [4,6] and polymeric foams (expanded polystyrene and polyurethane foams). These materials find widespread use in providing thermal insulation and soundproofing for both civil and industrial buildings [4,7]. However, conventional polymer-based foams are produced from primary raw materials, often derived from fossil fuels. Additionally, the release of fibers from mineral wool can raise health concerns [8]. In light of increased awareness of environmental and health issues, there is growing research interest in alternative materials. These materials aim to utilize secondary, renewable or recycled sources to meet sustainability and ecological requirements [5,7,9,10]. Rigid polyurethane (PU) foams are widely used as insulation materials in buildings, refrigeration and appliances [11]. They offer high thermal resistance, effectively reducing heat transfer and improving energy efficiency. Additionally, they provide strength, rigidity and dimensional stability. In the automotive industry, rigid PU foams are used for components such as seat cushions, headrests and interior trim, enhancing comfort, impact resistance and weight reduction. These foams are also employed in protective packaging due to their shock absorption and vibration-dampening properties. Furthermore, they find applications in the marine and aerospace industries owing to their high strength-to-weight ratios and resistance to water and chemicals [11,12]. While rigid PU foams have various benefits, their disposal and recycling presents challenges due to their thermosetting nature [13,14]. Unlike thermoplastic polymers that can be melted and reprocessed multiple times, thermoset polymers undergo irreversible crosslinking reactions that make them difficult to reshape or remold. As a result, traditional recycling methods used for thermoplastics, such as mechanical recycling through melting and reforming, are not effective. The current predominant methods for waste management of rigid PU involve landfilling or incineration. The latter option, in particular, can release toxic byproducts and pollutants into the environment, raising concerns regarding air pollution and human health. This highlights the need for alternative solutions in the effective management of PU waste. Efforts are being made to address this challenge by developing alternative recycling methods, including chemical recycling, depolymerization and conversion into bio-based materials. These approaches aim to recover valuable materials from PU waste, diverting it from landfills and promoting a more sustainable approach to polyurethane waste management [15,16,17,18]. The focus of this study is on a novel material produced using a circular economy approach. It involves incorporating rigid PU waste, ground into fine powder form, into a matrix based on scleroglucan (SG), a natural biopolymer. The objective is to produce a foam material with good thermal and acoustic insulation properties. To the best of our knowledge, low-temperature production methods without the use of blowing agents are not currently available for this type of material. In recent works, we successfully incorporated glass and fiberglass waste into an open-cell foam structure using alginate as the biopolymer [19,20,21]. However, we faced limitations in including other types of powdered waste, such as rigid PU powder, bricks or other stone-derived construction waste. This was due to the low density of the PU powder and the high density of bricks/stone waste. Additionally, the chemical activity of Ca^2+^, Mg^2+^ or Fe ions within bricks powder caused rapid and uncontrolled crosslinking of the alginate gel. In this study, we propose a similar approach but use a different bio-based polymer to overcome these challenges. Scleroglucan is a neutral and hydrosoluble β-1,3-β-1,6-glucan secreted by the filamentous fungus sclerotium [22,23,24]. Classified as a polysaccharide, it consists of a linear chain of glucose units connected by glycosidic bonds. SG is known to form highly viscous solutions with pseudoplastic non-Newtonian behavior, even at low concentrations. Beyond its rheological properties, SG exhibits excellent film-forming and gel-forming characteristics, making it suitable for various applications, including coatings, adhesives, films and drug-delivery systems [25,26]. SG-based hydrogels are formed by dispersing it in water to create a homogeneous solution. The unique molecular structure of SG allows it to absorb water and swell, leading to the expansion of chains, resulting in the formation of a viscous solution. As the concentration increases, polymer chains entangle, forming a three-dimensional network. Gelation occurs, transforming the solution into a hydrogel with structural stability that retains its shape [27,28,29,30]. The gelation process and the properties of the hydrogel can be influenced by factors such as the concentration of scleroglucan, temperature and the presence of other additives in the solution. SG solutions at concentrations of 0.2% *w*/*w* behave as entangled polymeric solutions, while solutions at higher concentrations, such as 1.0% and 2% *w*/*w*, behave as weak gels without the need for crosslinking agents [31]. This behavior allows for the formation of hydrogels without chemical modification, resulting in materials with unique properties. Furthermore, the subsequent removal of water via freeze-drying yields a low-density material comparable to an aerogel. Notably, SG gelation occurs more rapidly than that of alginate, and it does not require the use of chelating agents (such as Ca^2+^ ions). Moreover, SG allows for the effective incorporation of very light (PU) or very heavy (brick or rock) powders into the forming foams, whereas alginate is limited to medium-density powders (such as glass or fiberglass). Various molecules, including glycols and polyalcohols, are known to modify the mechanical properties of SG-based hydrogels in terms of strength and stiffness [27], while other molecules are used as binders or thickeners [28]. Polyvinylpyrrolidone (PVP), also known as povidone, is commonly used as binder in various pharmaceutical and biomedical applications, including hydrogels [32]. In brief, our synthesis route is based on the preparation of a hydrogel, which is subsequently freeze-dried to preserve its three-dimensional porous network. Freeze-drying removes the entrapped water during gelation, preventing the collapse of pores. This study successfully demonstrates the possibility of incorporating rigid PU waste as filler within the biopolymer network. We also explored the addition of different binders and plasticizers (PVP and glycerol, the latter already tested as a plasticizer with alginate-based gels) to fine-tune mechanical, thermal insulation and soundproofing properties. This innovative foam material effectively addresses the utilization of secondary and renewable raw sources while simultaneously tackling the issue of rigid PU waste disposal and recycling. Unlike mineral fiber-based materials currently available on the market, this foam mitigates the problem of fiber release, leading to significant environmental and human health benefits. With its favorable properties and a production process rooted in the principles of the circular economy, this novel material holds promise for thermal and acoustic insulation applications across various sectors.

## 2. Materials and Methods

### 2.1. Samples Production

Scleroglucan (SG, Sclerotium Gum, CS 11 QD) was purchased from Cargill. Glycerol (G, ≥99.5%) and polyvynylpyrrolidone (PVP, K30) were obtained from Sigma Aldrich (Merck KGaA, Darmstadt, Germany). Rigid polyurethane foam (PU) production scraps were freely donated by a company in northeastern Italy. These PU scraps underwent a mechanical grinding process, followed by screening and sieving to obtain a fine powder with particle sizes less than 200 μm. The production of foam was conducted using a sol-gel process, following partially a procedure previously documented [19,20,21]. In brief, a sol was prepared by mixing SG with water, PU powder and a plasticizer (PVP, glycerol or a combination of both—see Table 1 for composition details), obtaining a sol. This sol was then poured into molds measuring 200 × 200 mm^2^ (with a sol height of approximately 20 mm). The samples were left at room temperature for 30 min to complete the gelation process. After gelation, they were placed in a freezer at −20 °C for 24 h and subsequently subjected to a 48-h freeze-drying process using a 5Pascal LIO5P freeze dryer to remove water. At least three samples were produced for each composition. The initial reference sample composition (expressed as weight/volume %) was set as follows: SG = 1.5; PVP = 1.5; PU = 5. This composition was determined, following preliminary tests, to strike an optimal balance between maintaining a low sample density and maximizing its recycled content, mechanical stiffness and insulating properties. Variations in SG and PU powder concentrations were introduced to study their impact on the final properties, while different concentrations and ratios of PVP and glycerol were tested to assess their influence as binders and/or plasticizers. Samples 12, 13 and 14 were produced and tested for comparative purposes only. In these samples, the PU powder was replaced with powders derived from waste glass, brick and carbon fibers (5% weight/volume). This was done to demonstrate the feasibility of utilizing different types of powdered waste within the SG matrix as a proof of concept. Following measurement and weighing procedures (as detailed in Section 2.2), the samples were cut into smaller sizes. Square-based sample (150 × 150 × 15 mm^3^) were utilized for thermal conductivity measurements, while cylindrical samples (diameter 45 mm) were used for acoustic characterization and mechanical testing. Furthermore, commercial samples of rock wool, expanded polystyrene (EPS) and rigid PU foam were tested under the same conditions (refer to Table 2 and the method descriptions in the following paragraphs).

### 2.2. Dimension, Mass and Density Determination

Before conducting measurements and testing, all samples were conditioned for 24 h at 20 °C and 40% relative humidity. The dimensions of the dried samples were measured using a digital caliper (RS Pro, code 841-25), with values rounded to the nearest 10^−1^ mm, and averaging three measurements for each dimension. Three measurements were taken for each dimension, and the average was calculated. Mass measurements were performed using a digital balance (Sartorius CP244S, Sartorius AG, Göttingen, Germany), with values rounded to the nearest 10^−1^ g. For square-based samples, volume was calculated by multiplying the three dimensions, while for cylindrical samples, it was determined using the formula πR2H (with R representing the radius and H the height). Sample density, rounded to the nearest kg m^−3^, was then calculated by dividing the mass by the volume.

### 2.3. Macro and Micrographs

Macroscopic images of the samples were captured using a digital camera (Panasonic Lumix DMC-TZ80, Panasonic Marketing Europe GmbH, Wiesbaden, Germany). Scanning Electron Microscopy (SEM) images were obtained using a SUPRA40 SEM (Carl Zeiss Microscopy GmbH, Jena, Germany) operating at a 3 kV acceleration voltage with a secondary electron detector.

### 2.4. Mechanical Testing

Compression tests were conducted using a Shimadzu AGS-X (Shimadzu Europa GmbH, Duisburg, Germany) 10 dynamometer with a 10 kN load cell. Three cylindrical samples (diameter 45 mm; thickness 15 mm) were tested for each composition. The test speed was set to 1.5 mm min^−1^, while the signal acquisition time was set at 250 ms. Mechanical properties, including compression modulus and strength, were determined in accordance with ASTM C165 [33]. Compression strength was conventionally recorded at 25% and 50% strain for all samples to facilitate comparison.

### 2.5. Thermal Conductivity Determination

Thermal conductivity measurements were carried out using a Netzsch HFM 446 (NETZSCH-Feinmahltechnik GmbH, Selb, Germany) heat flow meter on square-based samples (150 × 150 × 15 mm^3^) in compliance with the ASTM C518 [34], at an average temperature of 25 °C.

### 2.6. Sound Absorption Measurements

To determine the sound absorption properties of the samples, a two-microphone plane wave impedance tube (Kundt’s tube) was employed, following the ISO 10534-2 standard [35]. The noise reduction coefficient (NRC) was calculated in accordance with the ASTM C423 [36]. Three cylindrical samples (diameter 45 mm; thickness 15 mm) were tested for each composition.

## 3. Results and Discussion

### 3.1. Foam Structure

**Figure 1:**
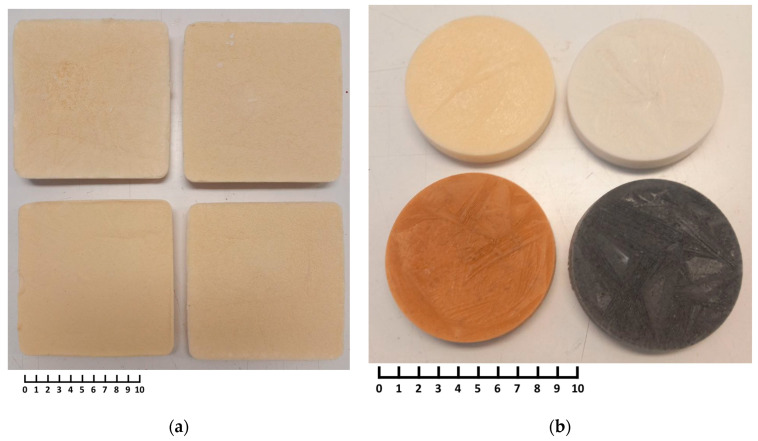
Representative images of foam samples: (**a**) obtained from PU powders; (**b**) obtained from PU (yellow sample), glass (white sample), brick (brown sample) and carbon fibers (black sample) powders (for comparison). Scale bars are in cm.

**Figure 2:**
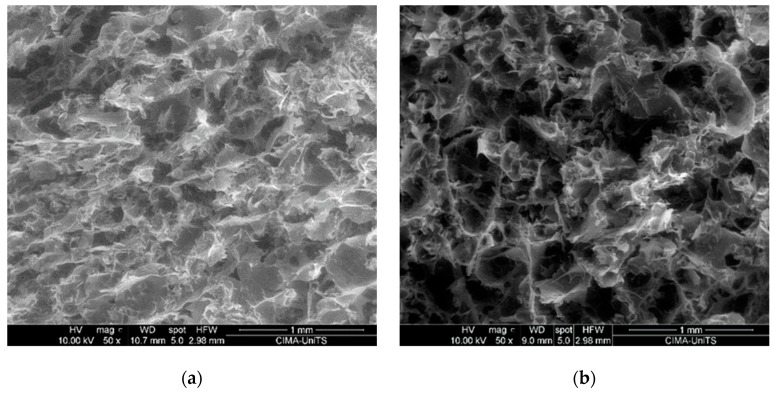

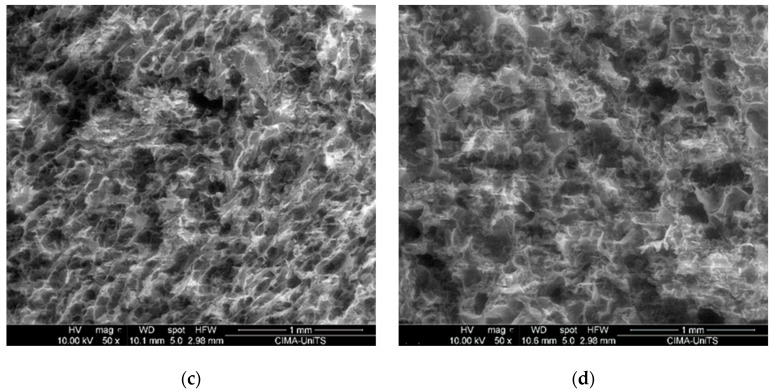
SEM images illustrating the microstructure of foam samples with varying compositions, expressed as weight/volume percentages: (**a**) Scleroglucan 1.5%, PVP 1.5% and PU 5%; (**b**) Scleroglucan 2%, PVP 1.5% and PU 5%; (**c**) Scleroglucan 1.0%, PVP 1.5% and PU 5%; (**d**) Scleroglucan 1.5%, PVP 1.5% and PU 15%.

Images of representative samples are shown in Figure 1, highlighting the visual aspects of the foam material. Figure 2 presents a representative SEM image, providing detailed surface morphology. The observed open-cell porous structure of the foam is essential for achieving the desired lightweight properties and insulation capabilities, as outlined in the subsequent paragraphs. Average pore size range from 50 to 100 μm. This structure is maintained through the freeze-drying process, which prevented pore collapse during the water removal.

### 3.2. Sample Density

The sample densities are provided in Table 2. In general, samples with the reference quantity of PU powder (5% wt./vol) exhibit densities ranging between 50 and 65 kg m^−3^. It is worth noting that samples containing glycerol (sample nr. 4, 6 and 7) are somewhat heavier than the others. This increased weight is likely attributed to moisture absorption from the atmosphere, as glycerol is a hygroscopic molecule. Samples numbered 2 and 3 (containing 10% and 15% wt./vol of PU powder, respectively) display higher densities, measuring 78 and 105 kg m^−3^, respectively, due to their higher solid fraction. In comparison, conventional mineral wools exhibit higher densities (100–150 kg m^−3^) while polymeric foams are lighter respect most of our samples, ranging from 15 to 20 kg m^−3^ [37,38].

### 3.3. Mechanical Properties

**Figure 3:**
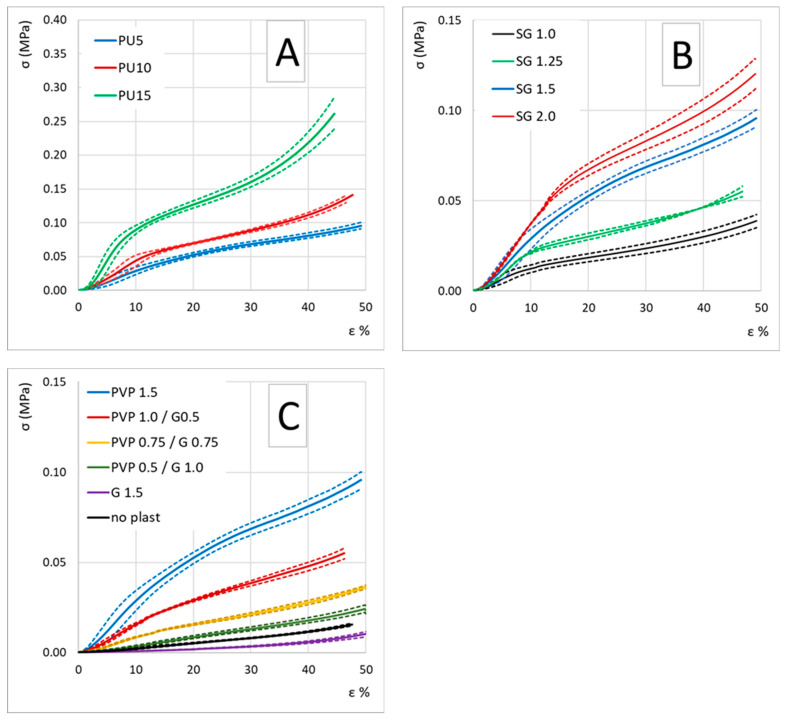
(**A**) Stress/strain compression curves for foam samples with varied PU amounts (5%, 10% and 15% wt./vol.); (**B**) stress/strain compression curves for foam samples with different scleroglucan (1.0%, 1.25%, 1.5% and 2.0% wt./vol.); (**C**) stress/strain compression curves for foam samples with different concentrations of PVP and Glycerol. Mean curves are represented by solid lines; standard deviation by dotted lines.

Figure 3 displays the average compression test stress-strain curves, labeled as A, B and C. In Figure 3A, we compare samples produced with varying concentrations of polyurethane (PU) expressed as a percentage of weight/volume in the initial aqueous suspension (PU5, PU10 and PU15). Similarly, Figure 3B compares samples produced with different concentrations of scleroglucan (SG) at 1.0%, 1.5% and 2.0% weight/volume. Figure 3C illustrates stress-strain curves for samples produced with varying concentrations of plasticizers (polyvynilpyrrolidone—PVP; and glycerol—G), denoted as “PVP x/G y”, where x and y represent the percentages of weight/volume in the initial aqueous suspension. Additionally, a stress-strain curve for samples produced without plasticizers, represented by the black curve, is shown. The values of compression modulus and compression strength, conventionally recorded at 25% and 50% strain, are reported in Table 2. Notably, the majority of the samples exhibit mechanical properties comparable to those of standard mineral wool [37]. However, these properties fall significantly short when compared to rigid PU foam and EPS foam. It is worth highlighting that only the sample containing 15% weight/volume of PU demonstrates a compression modulus and strength within the same range as EPS foam [38].

**Figure 4:**
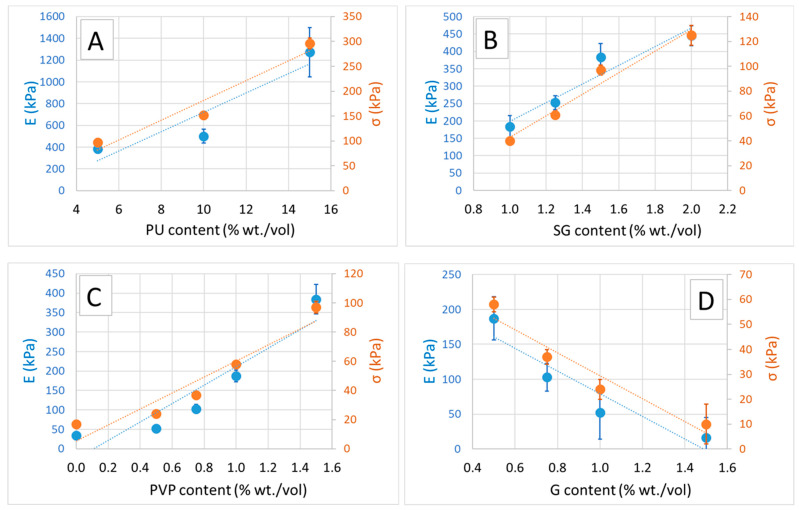
Relationship between Compression Modulus E (blue, primary y-axis) and Compression Strength (orange, secondary y-axis) with respect to the content (wt./vol.) of the following components: (**A**) Polyurethane Powder, (**B**) Scleroglucan, (**C**) Polyvinylpyrrolidone and (**D**) Glycerol.

The data suggests that both modulus and compressive strength tend to increase with higher PU, SG and PVP content, while they appear to decrease with the addition of glycerol. This effect of glycerol-induced stiffness reduction in alginate-based matrices is already known; a similar effect can be hypothesized in SG-based matrices [39,40,41]. A glycerol concentration of 1.5% weight/volume seems to dramatically decrease both compressive modulus and strength. Conversely, higher PVP content appears to enhance both modulus and strength. Trend of modulus E and compression strength as a function of sample composition are reported in Figure 4.

### 3.4. Sound Absorption Properties

**Figure 5:**
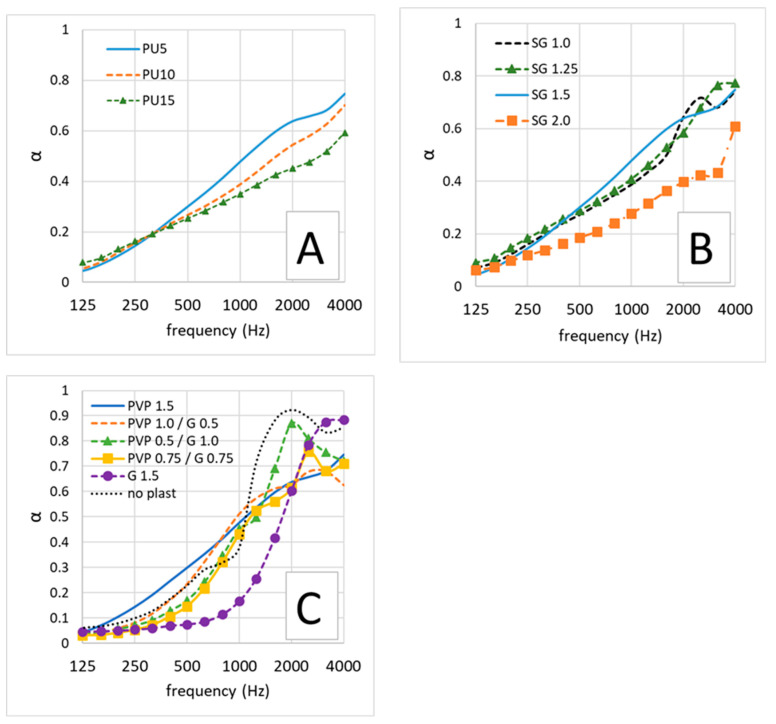
Sound-absorption coefficient curves of foam samples. (**A**) Comparison between samples produces with different amount of PU (5, 10 and 15 wt./vol %); (**B**) comparison between samples produced with different concentration of SG (1.0, 1.25, 1.5 and 2.0% wt./vol.); (**C**) comparison between samples produced with different concentrations of PVP and G.

Average noise absorption coefficient curves as a function of sound frequency are presented in Figure 5. In Figure 5A, a comparison is made between samples produced with different concentrations of PU (labeled as PU5, PU10 and PU15, with the number denoting the PU concentration as a percentage of weight/volume in the initial aqueous suspension). Similarly, compares samples produced with varying concentrations of SG (1.0%, 1.5% and 2.0% weight/volume). Figure 5C displays the curves for samples produced with different concentrations of plasticizer molecules (glycerol, PVP or no plasticizer). The values of the noise reduction coefficient (NRC) are detailed in Table 2.

The observed values and overall acoustic performance do not seem to follow a clear trend solely based on sample composition. However, it is noteworthy that samples containing glycerol exhibit superior noise absorption capabilities. The open pore structure of the material facilitates the dissipation of sound waves. As sound waves penetrate the material, they encounter the irregular surfaces and porous structure, causing multiple reflections and interactions. This results in the dissipation of acoustic energy within the material due to air resonance, damping and multiple reflection [42,43]. Softer materials tend to absorb sound more effectively due to the deformation that occurs when sound waves interact with the material’s structure [42,43]. The NRC values are either on par with or even surpass those of commercially available sound-absorbing materials.

### 3.5. Thermal Insulation Properties

The values of thermal conductivity (reported in Table 2) fall within the range of 36 to 38 mW m^−1^ K^−1^. Interestingly, these values do not seem to exhibit a clear correlation with the density of the samples. For instance, samples with a density of 48 kg m^−3^ exhibit a thermal conductivity of 36.5 mW m^−1^ K^−1^, while those with a density of 94 kg m^−3^ have a slightly higher thermal conductivity of 37.8 mW m^−1^ K^−1^. Notably, the sample with the highest thermal conductivity (41.2 mW m^−1^ K^−1^, with density of 42.2 kg m^−3^) is the one produced with 1.5% weight/volume glycerol, suggesting a higher water content in this particular sample due to moisture absorption. The variations in SG, PU or PVP concentration do not appear to significantly impact thermal conductivity. However, an increase in glycerol concentration seems to have a detrimental effect on the thermal insulation properties. For comparison, conventional materials such as expanded polystyrene or polyurethane foam typically exhibit slightly better thermal conductivity values in the range of 20–30 mW m^−1^ K^−1^ with respect to our samples, while the conductivity of mineral wool (35–37 mW m^−1^ K^−1^) lies in the same range [4,6,38,44]. It is worth noting that thermal conductivity is influenced by factors such as density and porosity structure. The thermal insulation mechanism involves minimizing heat conduction through reduced thermal conductivity, trapped air or gas acting as an insulator and potential reflection of thermal radiation. The majority of thermal conduction occurs in the solid phase, which is related to the sample density and contributes the most to the overall thermal conductivity [41]. In closed-cell materials, thermal insulation is favored due to reduced convective heat transfer, whereas in open-cell structures like ours, conduction in the gas phase plays a larger role, hindering optimal heat insulation performance [45]. Open-cell structure, coupled with a higher density compared to expanded polymeric foams, appears to limit its potential for achieving optimal heat insulation performance.

## 4. Conclusions

In this study, we successfully developed a lightweight thermal and acoustic insulation material by incorporating rigid PU waste into renewable biopolymers. This novel material exhibits an open porous structure, with densities ranging from 170 to 320 kg m^−3^, thermal conductivities between 35 and 65 mW m^−1^ K^−1^ and a noise-reducing factor within the range of 0.25–0.45. The compressive strengths as high as 1 MPa make it an interesting candidate for applications requiring a good combination of low weight, thermal/acoustic insulation and a minimal amount of mechanical strength.

Preliminary tests have shown that our innovative foam possesses insulation properties akin to those of typical mineral wool (<40 mW m^−1^ K^−1^). While its load-bearing capacity may not outshine that of certain polymeric rigid foams, our material surpasses polymeric foams of similar density in terms of soundproofing and thermal insulation properties.

Additionally, preliminary findings indicate that most of these properties can be finely adjusted by varying the biopolymer concentration and/or incorporating polysaccharides as plasticizers. Our approach prioritizes environmental sustainability by reducing energy consumption, limiting primary raw material usage and diverting rigid PU waste from landfills. As the industry increasingly emphasizes resource efficiency and environmental responsibility, this method aligns with these goals.

However, further research is needed to optimize the material’s properties, especially in terms of achieving optimal heat insulation performance. Additionally, long-term durability and environmental impact require deeper investigation.

Regarding the recyclability, compostability and biodegradability of the products, our findings indicate that the products are recyclable through mechanical grinding. This process allows for the recovery of powders that can be reprocessed in a manner similar to the original polyurethane (PU) powders, thereby supporting a circular economy approach to waste management. However, it is important to note that the products are not compostable or biodegradable due to the presence of rigid PU powder. While the scleroglucan component alone may possess compostable and biodegradable properties, it is challenging to separate from the PU after processing. As a result, the overall material cannot be considered compostable or biodegradable in its current form.

Our research underscores the importance of considering the environmental implications of materials throughout their lifecycle, from production to end-of-life disposal. While our products offer recyclability benefits, future studies may explore methods to enhance the environmental sustainability of composite materials containing both synthetic and biopolymer components. In conclusion, this study presents a significant step towards a more sustainable and eco-friendly future in insulation materials, providing a foundation for future research and development in the quest for innovative environmentally friendly insulation solutions.

## Figures and Tables

**Table 1:** Composition of samples.

Sample Nr.	SG (% wt./vol.)	PVP (% wt./vol.)	G (% wt./vol.)	Filler (% wt./vol.)	Filler Type
1	1.5	1.5	-	5	PU powder
2	1.5	1.5	-	10	PU powder
3	1.5	1.5	-	15	PU powder
4	1.5	0.75	0.75	5	PU powder
5	1.5	1.00	0.50	5	PU powder
6	1.5	0.50	1.00	5	PU powder
7	1.5	-	1.50	5	PU powder
8	1.5	-	-	5	PU powder
9	1.00	1.5	-	5	PU powder
10	1.25	1.5	-	5	PU powder
11	2.00	1.5	-	5	PU powder
12	1.5	1.5	-	5	glass powder
13	1.5	1.5	-	5	brick powder
14	1.5	1.5	-	5	carbon fibers powder

**Table 2:** Properties of samples: debsity (ρ), compression modulus (E), compression strength (σc), noise reduction coefficient (NRC) and thermal conductivity (λ). The values (*) measured on commercial samples of rock wool, expanded polystyrene (EPS) and rigid PU foam are reported for comparison.

Sample Nr.	Density (kg m^−3^)	E (kPa)	σ_c_ at ε = 25% (kPa)	σ_c_ at ε = 50% (kPa)	NRC	λ (mW m^−1^ K^−1^)
1	55.2 ± 0.5	384 ± 38	61 ± 3	97 ± 4	0.40	36.5 ± 1.0
2	77.5 ± 1.4	500 ± 64	79 ± 2	152 ± 6	0.35	36.7 ± 1.5
3	105.4 ± 1.4	1273 ± 226	138 ± 4	296 ± 11	0.30	38.8 ± 1.3
4	58.8 ± 0.6	103 ± 11	19 ± 1	37 ± 1	0.40	36.9 ± 0.5
5	50.8 ± 0.8	187 ± 15	33 ± 0	58 ± 1	0.45	36.8 ± 2.0
6	55.7 ± 1.1	52 ± 3	11 ± 1	24 ± 2	0.40	37.2 ± 1.4
7	65.9 ± 1.6	16 ± 1	3 ± 0	10 ± 1	0.30	36.0 ± 0.6
8	42.5 ± 2.5	34 ± 3	7 ± 0	17 ± 1	0.45	41.2 ± 1.1
9	50.5 ± 1.1	184 ± 31	21 ± 1	40 ± 1	0.35	36.7 ± 1.5
10	50.7 ± 0.5	253 ± 10	34 ± 1	61 ± 1	0.40	36.5 ± 1.2
11	57.9 ± 1.1	445 ± 29	76 ± 9	125 ± 8	0.25	36.6 ± 1.0
12	60.5 ± 2.9	270 ± 31	38 ± 1	64 ± 1	0.35	-
13	62.2 ± 1.6	228 ± 10	35 ± 0	58 ± 1	0.30	-
14	74.8 ± 3.4	270 ± 10	50 ± 1	80 ± 0	0.30	-
rock wool *	151 ± 5.0	500 ± 20	60 ± 1	80 ± 1	0.30	37.7 ± 0.5
EPS *	18.5 ± 0.6	≈ 2.0 × 10^3^	120 ± 3	180 ± 5	0.20	36.7 ± 2.5
Rigid PU *	52.1 ± 0.7	≈ 1.1 × 10^4^	410 ± 10	470 ± 20	0.05	42.0 ± 1.2

## Data Availability

Data are contained within the article. The raw data supporting the conclusions of this article will be made available by the authors on request.
